# Deciphering the Molecular Mechanisms Sustaining the Estrogenic Activity of the Two Major Dietary Compounds Zearalenone and Apigenin in ER-Positive Breast Cancer Cell Lines

**DOI:** 10.3390/nu11020237

**Published:** 2019-01-22

**Authors:** Sylvain Lecomte, Florence Demay, Thu Ha Pham, Solenn Moulis, Théo Efstathiou, Frédéric Chalmel, Farzad Pakdel

**Affiliations:** 1 Univ Rennes, Inserm, EHESP, IRSET (Institut de Recherche en Santé, Environnement et Travail)-UMR_S1085, F-35000 Rennes, France; sylvain.lecomte35@gmail.com (S.L.); florence.demay@univ-rennes1.fr (F.D.); thu-ha.pham@univ-rennes1.fr (T.H.P.); solenn.moulis@univ-rennes1.fr (S.M.); frederic.chalmel@univ-rennes1.fr (F.C.); 2 Laboratoire Nutrinov, Technopole Atalante Champeaux, 8 rue Jules Maillard de la Gournerie, 35012 Rennes CEDEX, France; theo.efstathiou@nutrinov.com

**Keywords:** dietary compounds, estrogen receptor, breast cancer, gene expression, endocrine disruption

## Abstract

The flavone apigenin and the mycotoxin zearalenone are two major compounds found in the human diet which bind estrogen receptors (ERs), and therefore influence ER activity. However, the underlying mechanisms are not well known. To unravel the molecular mechanisms that could explain the differential effect of zearalenone and apigenin on ER-positive breast cancer cell proliferation, gene-reporter assays, chromatin immunoprecipitation (ChIP) experiments, proliferation assays and transcriptomic analysis were performed. We found that zearalenone and apigenin transactivated ERs and promoted the expression of estradiol (E2)-responsive genes. However, zearalenone clearly enhanced cellular proliferation, while apigenin appeared to be antiestrogenic in the presence of E2 in both ER-positive breast cancer cell lines, MCF-7 and T47D. The transcriptomic analysis showed that both compounds regulate gene expression in the same way, but with differences in intensity. Two major sets of genes were identified; one set was linked to the cell cycle and the other set was linked to stress response and growth arrest. Our results show that the transcription dynamics in gene regulation induced by apigenin were somehow different with zearalenone and E2 and may explain the differential effect of these compounds on the phenotype of the breast cancer cell. Together, our results confirmed the potential health benefit effect of apigenin, while zearalenone appeared to be a true endocrine-disrupting compound.

## 1. Introduction

The human population is commonly exposed to numerous estrogenic compounds through the environment, including the diet. Epidemiological studies estimate that exposure to these environmental chemicals may potentially impact human and animal health [1]. These approaches provided statistical associations between environmental estrogens and fertility or various cancers, particularly breast cancer [2]. Some of these statistical associations, notably, the association with soy consumption, showed beneficial effects [3,4]. Among these environmental estrogens, some are chemicals such as pesticides or insecticides; others are naturally produced by plants or fungi. This is the case with the well-documented flavones, isoflavones, stilbenes, mycotoxins, and so on. In a previous study, we screened the impact of different compounds produced by plants and belonging to different chemical classes on two cellular phenotypes, namely, cellular proliferation and cellular differentiation. From this screen, we identified zearalenone and apigenin, which had opposite biological effects. Indeed, we had shown that zearalenone and apigenin were able to transactivate ERs in different cell models and regulated ER-gene expression such as chemokine (C-X-C motif) ligand 12 (CXCL12). Moreover, zearalenone was found as a strong inducer of cellular proliferation and toxic for neuronal differentiation while apigenin was a weak cellular proliferation inducer and a good potentiator of neuronal differentiation [5]. 

Zearalenone is a mycotoxin mainly produced by Fusarium fungi as a second metabolite and is reported to have adverse effects on health. Particularly, zearalenone has been associated with cancer, immunotoxicity, reprotoxicity, and advanced puberty, probably via its interaction with estrogen receptors (ERs) [6]. Because zearalenone is found in corn, wheat, barley, sorghum and rye, the human population is exposed directly through the consumption of contaminated cereals or indirectly through livestock fed contaminated cereals. Moreover, zearalenone is resistant to food processing methods such as heating. Currently, the advisory level for zearalenone is not defined in the USA, while it is limited to 20–100 µg/kg for cereals and up to 400 µg/kg for maize in Europe, with a tolerable daily intake of 0.25 µg/kg body weight. A recent work assessed the level of circulating zearalenone and its metabolites in a group of 48 women. Free zearalenone was detected in serum from 85.4% of the participants, with a mean concentration of 0.026 ± 0.022 ng/mL [7].

Unlike zearalenone, the flavone apigenin, which is found in numerous plant foods, is described as having beneficial effects on human health, particularly on cancer [8]. Indeed, apigenin is present in teas and dry herbs, particularly chamomile flowers (395–1200 mg/100 g dry weight) and parsley (up to 1500 mg/100 g fresh weight), and in several other foods such as juices, fruits, honey and cereals [9]. It was estimated that women in the Nurses’ Health Study ingested up to 1.3 mg/day of apigenin [10]. Pharmacokinetics studies were performed using aglycone form of apigenin. For instance, after oral administration of radiolabeled apigenin or the flower extract of *Daphne genkwa*, a plasma concentration peak was obtained after 1 h and reached approximately 40–50 ng/mL in rats. Moreover, these studies showed the sustained persistence of apigenin, suggesting an accumulation in tissues [11,12]. However, in humans, after consumption of parsley or chamomile extract, the plasma concentration of apigenin remains low (<1 µM), possibly due to its lower bioavailability in humans than in rodents and different metabolism by the intestinal microbiota [9,13]. Like zearalenone, apigenin binds to ERs and is able to transactivate them [5]. 

ERs belong to the nuclear receptor superfamily and are encoded by two distinct genes. ERα is the main regulator of estrogen-dependent genes. ERβ, when it is coexpressed with ERα, has a tendency to restrain ERα activity [14]. In cells, ERs bind directly to DNA on estrogen-responsive elements (EREs) or interact with other transcription factors, such as specificity protein 1 (SP1) or activator protein 1 (AP1). They are also able to modulate intracellular pathways such as the mitogen-activated protein kinase (MAPK) and phosphatidylinositol 3-kinase (PI3K)/protein kinase B (AKT) pathways. ERs are extensively distributed in both male and female organisms, where they exert pleiotropic effects [15,16]. Estradiol (E2) is the natural hormone that binds ERs and is involved in the development and maintenance of the sexual organs in women. For instance, E2 is involved in breast epithelial cell proliferation and survival and, unfortunately, in breast cancer initiation and growth [17]. Breast cancer is the most prevalent cancer in women worldwide, with an incidence of approximately 90 cases per 100,000 women in developed countries. Breast cancer is ranked by the WHO as the fifth overall cause of death from cancer (522,000 deaths) [18]. ER-positive breast cancer represents approximately 80% of cases; therefore, ERs, particularly ERα, are considered good prognostic factors and are prime targets for therapy.

The aim of this study was to examine ER-mediated activation of cell proliferation by zearalenone and apigenin and to try to decipher mechanisms that sustained the differential activity of these compounds. In our previous work [5], we have used two ER-positive breast cancer cell lines (MCF-7 and T47D) to study the proliferative effect of these compounds. The results showed that the effect of zearalenone and apigenin was quite similar in these two cell lines. Zearalenone showed full estrogenic activity by stimulating cell proliferation, while apigenin exhibited partial agonistic activity, increasing cell numbers slightly. Thus, in this study, we confirmed our previous data on MCF-7 and T47D cell proliferation and showed the combined effect of zearalenone and apigenin with estradiol. However, the mostly of molecular analysis were performed on the MCF-7 cell line which is the main representing of luminal A breast cancer [19]. Luminal A and luminal B breast cancer belong to the ER-positive breast cancer family, but luminal A possesses more ERα than luminal B. Consequently, it is a reasonable model to study the estrogenic activity of compounds. We confirmed that both compounds activate ERα and ChIP assays on different ER binding sites clearly indicated that the actions of apigenin and zearalenone did not reside in the ability of these compounds to induce ER-DNA binding. However, these compounds have distinct effects on proliferation. By transcriptomic analysis, we found that the differential regulation of genes in response to zearalenone treatment perfectly mimicked the response to E2, while apigenin induced a distinct set of transcriptional response genes. More precisely, compared to gene transcription in response to E2 and zearalenone, the transcription of E2-target genes involved in cell growth was much less stimulated by apigenin, and, inversely, the transcription of those involved in cell growth arrest and apoptosis were much more stimulated (or less repressed) by apigenin.

## 2. Materials and Methods

### 2.1. Cell Culture and Reagents

MCF-7 cells were obtained from Jérôme Eeckhoute (Dana Farber Cancer Institute) and T47D cells were purchased from the American Type Culture Collection (Manassas, VA, USA). Cells were maintained in Dulbecco’s Modified Eagle Medium (DMEM) (MCF-7) or Roswell Park Memorial Institute (RPMI) 1640 Medium (T47D) containing 4.5 g/L glucose supplemented with nonessential amino acids, penicillin/streptomycin (Invitrogen) and 10% fetal bovine serum (FBS) (Biowest) at 37 °C under 5% CO_2_. For steroid treatment, cells were cultured for at least 24 h in steroid- and serum-deprived DMEM without phenol red and with 1.5% charcoal/dextran-stripped FBS (Biowest). Estradiol (E2), apigenin, and zearalenone were purchased from Sigma Aldrich (St Louis, MI, USA). ICI_182,780_ was purchased from Tocris Bioscience (Bristol, UK).

### 2.2. Luciferase Assay

First, 50,000 cells per wells were plated in 24-well multiwell plates. After serum and steroid deprivation, cells were transfected overnight with 100 ng of an estrogen responsive element-thymidine kinase (ERE-TK)-luciferase vector, which codes for luciferase under the control of one ERE, and with 50 ng of a cytomegalo virus (CMV)-β galactosidase vector, which served as a transfection efficiency control, using JetPEI (Polyplus Transfection, Illkrich, France). Next, cells were treated with solvent as a control, 10^−9^ M E2 or various doses of apigenin or zearalenone for different time periods. ICI_182,780_ at a concentration of 10^−6^ M was used as an ER inhibitor to test the specificity of ER transactivation. Cells were lysed in Passive Lysis Buffer (Promega, Madison, WI, USA), and luciferase activity was determined using a luciferase assay system (Promega, Madison, WI, USA).

### 2.3. Chromatin Immunoprecipitation

Cells were treated with solvent as a control, with 10^−9^ M E2, 10^−8^ M zearalenone or 10^−5^ M apigenin for 1 h and chromatin immunoprecipitation was performed as described in supplementary files of [20]. In this study the anti-ERα (HC20, Santacruz, Dallas, TX, USA) was used.

An enrichment analysis of the proximal ERE of GREB1 (Fwd: CACTTTGAGCAAAAGCCACA and Rev: GACCCAGTTGCCACACTTTT), on the enhancer of CUE domain containing 1 (CUEDC1) (Fwd: AGCATTGGTAAGGTCAGGCT and Rev: AGGAAGCTGGTGTCTTGGC), on enhancer 1 of x-box binding protein 1 (XBP1) (Fwd: TCACAGGCTGCCAAGTATCT and Rev: TGGCGTAATTCAAACCCTGC) and on enhancer 2 of XBP1 (Fwd: AAACAATAGCCCAGAAGCCG and Rev: AGTCCAAGGGCACATTCTCA) was performed by real-time polymerase chain reaction PCR on a CFX384 Touch system (Bio-Rad) with 2 µL of immunoprecipitation product or 0.2 µL of input DNA, 500 nM primers, and iTaq Universal SYBR Green Supermix (Bio-Rad, Hercules, CA, USA).

### 2.4. RNA Extraction and Real-Time PCR

Cells were treated with solvent as a control, with 10^−9^ M E2 or with various concentrations of apigenin or zearalenone as indicated in the legend figures. RNA extraction and real time PCR were performed as described in [20]. The sequence of the primers used in this study are described in Table 1.

### 2.5. Proliferation Assay

A total of 20,000 cells per well were plated in 24-well multiwell plates. Then, cells were deprived of steroids and serum for 72 h. Cells were treated for 6 days with doses of apigenin ranging from 10^−8^ M to 10^−5^ M or with doses of zearalenone ranging from 10^−11^ M to 10^−6^ M, with or without 10^−9^ M E_2_, with retreatment on day 3. After treatment, cells were trypsinized, and the cell number was determined with a TC10 Automated Cell Counter (Bio-Rad).

### 2.6. Cell Cycle Analysis

A total of 1,000,000 cells per dish were plated in 10 cm dishes. Then, cells were deprived of steroids and serum for 72 h. Cells were treated with solvent as a negative control, 10^−9^ M E2 as a positive control, 10^−5^ M apigenin or 10^−8^ M zearalenone for 72 h. After treatment, cells were trypsinized and fixed with 70% ethanol before being stained with propidium iodide (Sigma) in the presence of RNase A. The cell cycle was analyzed with a FACSCalibur flow cytometer (BD Biosciences, Franklin Lakes, NJ, USA).

### 2.7. Apoptosis Analysis

MCF-7 cells (4000 cells/well) were plated in 96-well plates. After 72 h of serum and steroid deprivation, the cells were treated for 72 h with solvent as a control, 10^−9^ M E2, 10^−5^ M apigenin or 10^−8^ M zearalenone. Terminal deoxynucleotidyl dUTP nick end labeling (TUNEL) staining was assessed with an In Situ Cell Death Detection Kit, Fluorescein (Roche) according to the manufacturer’s instructions. The fluorescence intensity and percentage of TUNEL-positive cells were determined with an ArrayScan VTI system (Thermo Fisher Scientific, Waltham, MA, USA) on the ImPACcell platform (Rennes, France).

### 2.8. Statistical Analysis

One-way analysis of variance (ANOVA) with Dunnett’s post hoc test comparing the control treatment to the other treatments was performed with GraphPad Prism software (version 5, GraphPad software, San Diego, CA, USA). Differences were considered significant at a *p*-value < 0.05.

### 2.9. Transcriptomic Analysis

Cells were treated for 24 h with solvent as a control, 10^−9^ M E2, 10^−5^ M apigenin or 10^−8^ M zearalenone RNA extraction, control quality, reverse transcription, labeling and spotting were performed as described in [20]. Sample hybridization, microarray scanning and data extraction were performed by the GeT-Biopuces Platform in Toulouse, France.

### 2.10. Microarray Data Analysis and Gene Filtration

Data analysis was performed using the Annotation, Mapping, Expression and Network (AMEN) suite of tools [21]. Briefly, probes showing a signal higher than a given background cutoff (corresponding to the overall median of the normalized dataset, 5.20) and at least a 1.5-fold change between the control and treatment signals were selected. To define a set of 3560 transcripts displaying significant statistical changes across comparisons, the linear models for microarray data (LIMMA) package was used (*F*-value adjusted with the false discovery rate method, *p* ≤ 0.05) [22]. The resulting probes were then partitioned into 6 expression clusters (termed C1-C6) using the hierarchical classification on principal component (HCPC) function implemented in the FactoMineR package [23].

### 2.11. Functional Data Mining

The enrichment analysis module implemented in the AMEN suite of tools [21] was used to identify biological processes significantly associated with each expression pattern by calculating Fisher’s exact probability using the Gaussian hypergeometric function (FDR-adjusted *p*-value ≤ 0.01, number of probes in a given group associated with a given annotation term ≥5).

### 2.12. Regulatory Network Analysis

Protein-gene regulation data were downloaded from the Transcription Factor Encyclopedia database [24]. A network representation showing all known protein-gene interactions between transcripts differentially expressed in the current project was drawn using AMEN.

## 3. Results

### 3.1. ER Is Transactivated by Zearalenone and Apigenin

**Figure 1:**
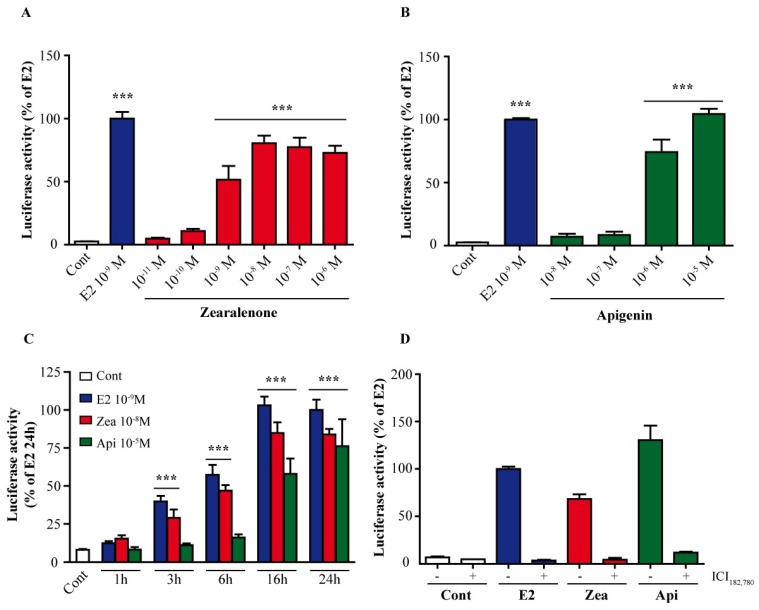
Effect of zearalenone and apigenin on estrogen receptor (ER) activation. MCF-7 cells were transfected with an estrogen-responsive element-thymidine kinase (ERE-TK)-luciferase reporter plasmid and a cytomegalo virus (CMV)-β galactosidase plasmid as a control for transfection efficiency. Then, cells were treated with solvent as a negative control (white), 10^−9^ M E2 as a positive control (blue) or various doses of zearalenone (red) (**A**) or apigenin (green) (**B**) for 24 h. The results are expressed as the percentage of luciferase activity attained with E2 treatment and are the means ± standard error of the mean (SEM) of three to four independent experiments. Cells were treated with solvent as a negative control (white), 10^-9^ M E2 as a positive control (blue), 10^−8^ M zearalenone (red) or 10^−5^ M apigenin (green) for 1 h, 3 h, 6 h, 16 h and 24 h (**C**). The results are expressed as the percentage of luciferase activity attained with E2 treatment at 24 h and are the means ± SEM of three independent experiments. (**D**) To confirm the estrogenic effects of apigenin and zearalenone, transfected cells were cotreated with 10^−6^ M ICI_182,780_ and either 10^−9^ M E2 (blue) or 10^−8^ M zearalenone (red) or 10^−5^ M apigenin (green). *** indicates a *p*-value < 0.001 by one-way analysis of variance (ANOVA) followed by Dunnett’s post hoc test for comparison of the control treatment with the other treatments.

To test optimal concentrations of zearalenone and apigenin, we first tested the transactivation of ERs in response to different concentrations of these compounds in ER-positive MCF-7 breast cancer cells with a luciferase reporter assay. Cells were transfected with a vector coding for luciferase under the control of an ER-responsive element (ERE) and were treated with various doses of zearalenone (Figure 1A) or apigenin (Figure 1B). Both compounds were able to significantly transactivate ERs at concentrations starting at 10^−9^ M zearalenone (*p* < 0.01) and 10^−6^ M apigenin (*p* < 0.01), as shown by the increase in luciferase activity. At 10^−5^ M apigenin, luciferase activity reached the same level observed for treatment with 10^−9^ M E2. The maximal activation with zearalenone was observed at 10^−8^ M. To examine the time-dependent activation of ERs, transfected cells were treated with 10^−9^ M E2, 10^−8^ M zearalenone or 10^−5^ M apigenin for 1 h, 3 h, 6 h, 16 h and 24 h (Figure 1C). In the presence of E2 and zearalenone, the activation profile of the luciferase reporter gene was similar. Both E2 and zearalenone stimulated luciferase activity after 3 h of treatment, whereas apigenin induced substantial luciferase activity after 16 h of treatment. Nevertheless, all three compounds similarly stimulated luciferase activity at 24 h, which was therefore used as the treatment time for the next experiments. 

To verify that the observed luciferase activity was ERα-dependent, cells were treated with 10^−9^ M E2, 10^−8^ M zearalenone or 10^−5^ M apigenin with or without 10^-6^ M of the antiestrogen ICI_182,780_ (Figure 1D). For E_2_, apigenin and zearalenone treatment, the luciferase activity was clearly reduced with ICI_182,780_ compared with that without ICI_182,780_, thus validating the transactivation of ERs by these molecules.

### 3.2. Zearalenone and Apigenin Are Able to Induce the Recruitment of ERα DNA-Binding at Chromatin Sites

**Figure 2:**
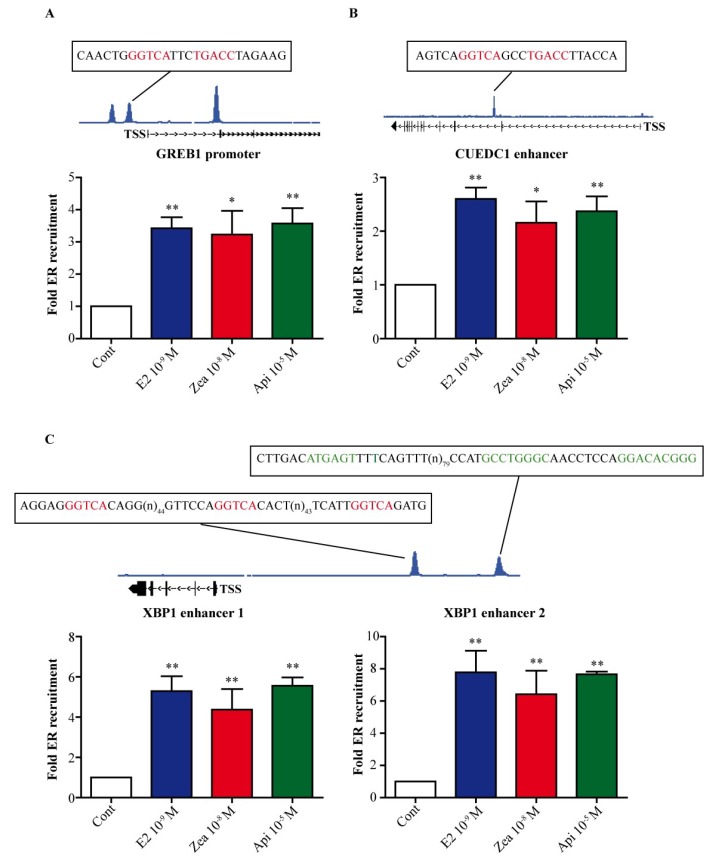
ERα recruitment to chromatin at distinct ER binding sites in response to E2, zearalenone and apigenin. MCF-7 cells were treated with solvent (white) as a negative control, 10^−9^ M E2 (blue) as a positive control, 10^−8^ M zearalenone (red) or 10^−5^ M apigenin (green) for 1 h. The recruitment of ERα to the GREB1 promoter (**A**), CUE domain containing 1 (CUEDC1) enhancer (**B**) and the two enhancers of x-box protein 1 (XBP1) (**C**) was assessed by chromatin immunoprecipitation followed by real-time PCR. For each binding site tested, the DNA sequence is indicated. The results are expressed in fold recruitment compared to control and are the means of four independent experiments. * indicates a *p*-value < 0.05 and ** indicates a *p*-value < 0.01 by one-way ANOVA followed by Dunnett’s post hoc test for comparison of the control treatment with the other treatments.

To test whether the observed differential kinetics between zearalenone and apigenin were due to a change in the recruitment of ERα to chromatin, we performed ChIP-PCR analysis on 4 distinct sites after 1 h of treatment with 10^−9^ M E2, 10^−8^ M zearalenone or 10^−5^ M apigenin (Figure 2). The binding sites studied were the proximal promoter of GREB1, which contains a consensus ERE sequence (Figure 2A), the superenhancer of CUEDC1, which also contains an ERE sequence but is localized in the core of the gene [25] (Figure 2B), and the two binding sites in the XBP1 enhancer (Figure 2C). The first contains 3 half-ERE binding sites, while the second contains one AP-1 and one SP-1 binding site. For all binding sites studied, both zearalenone and apigenin were able to significantly induce the recruitment of ERα as E2 did. This result indicates that the differential activation of ER target genes by apigenin versus that induced by E2 and zearalenone likely occurs at the transcriptional level and not at ER binding sites.

### 3.3. Induction of E2-Dependent Genes Is Different between Zearalenone and Apigenin 

**Figure 3:**
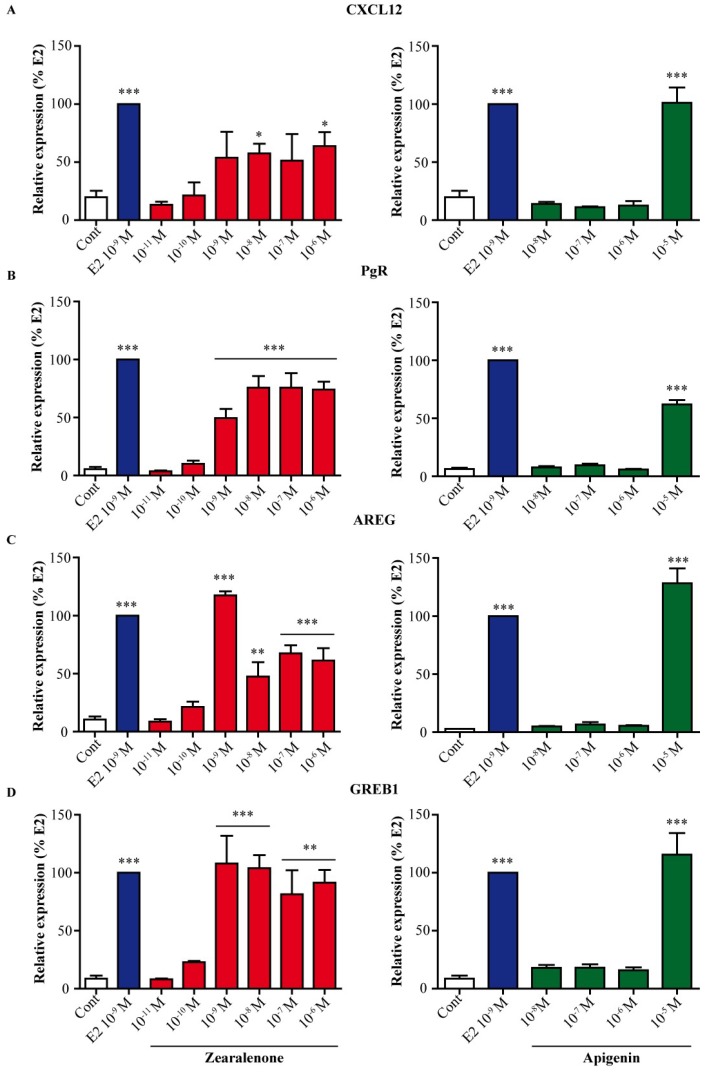
Effect of zearalenone and apigenin on the expression of endogenous E2 target genes. MCF-7 cells were treated with solvent (white) as a negative control, 10^−9^ M E2 (blue) as a positive control or with various doses of zearalenone (red) or apigenin (green) for 24 h. The expression of CXCL12 (**A**), PgR (**B**), AREG (**C**) and GREB1 (**D**) was assessed by real-time PCR. The expression level of each gene was normalized to the expression levels of the housekeeping genes GAPDH and TBP. The results are expressed as the percentage of the relative expression of transcripts obtained in E2-treated cells and are the means ± SEM of three independent experiments. * indicates a *p*-value < 0.05, ** indicates a *p*-value < 0.01, and *** indicates a *p*-value < 0.001 by one-way ANOVA followed by Dunnett’s post hoc test for comparison of the control treatment with the other treatments.

As zearalenone and apigenin activated ERs, we tested the potential effect of these molecules on the expression of endogenous E2-dependent genes, such as the chemokine CXCL12, the progesterone receptor (PgR), amphiregulin (AREG) and growth regulation in breast cancer 1 (GREB1) (Figure 3). MCF-7 cells were treated for 24 h with 10^−9^ M E2 as a positive control and with various doses of zearalenone (Figure 3, left) or apigenin (Figure 3, right). Although both compounds induced E2-dependent gene expression, marked dose-response differences were observed between zearalenone and apigenin. Indeed, zearalenone treatment resulted in an increase in gene expression starting at 10^−10^ M; this increase became significant compared to that the gene expression in the control cells at 10^−9^ M for PgR, AREG and GREB1 and at 10^−8^ M for CXCL12. Only the highest tested dose of apigenin induced a significant change in gene expression that reached the same level as produced by E2 treatment for any tested gene except for PgR, for which the effect reached only 60% of that induced by E2. Furthermore, the effect of zearalenone and apigenin on the expression of CXCL12 gene was examined in T47D (Figure S1A). Interestingly, zearalenone was more potent than apigenin in inducing CXCL12 gene expression.

### 3.4. Zearalenone and Apigenin Have Different ER-Dependent Proliferative Effects on MCF-7 Cells

**Figure 4:**
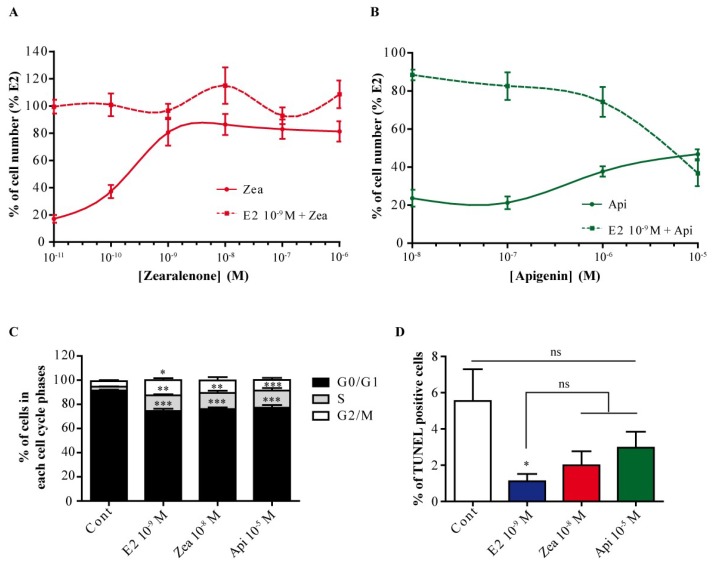
Effect of zearalenone and apigenin on MCF-7 proliferation. MCF-7 cells were treated with various doses of zearalenone (**A**) or apigenin (**B**) alone (solid lane) or in combination with 10^−9^ M E2 (dotted line) for 6 days, and the cell number was determined by counting. The results are expressed as the percentage of the cell numbers counted after E2 treatment and are the means ± SEM of three to four independent experiments. For the cell cycle (**C**) and apoptosis (**D**) assays, MCF-7 cells were treated for 3 days with 10^−9^ M E2, 10^−8^ M zearalenone or 10^−5^ M apigenin. The cell cycle was analyzed by flow cytometry after propidium iodide staining. The results are expressed as the percentage of cells in each phase of the cell cycle and are the means ± SEM of three to four independent experiments. Apoptosis analysis was performed by a terminal deoxynucleotidyl dUTP nick end labeling (TUNEL) assay, and the percentage of apoptotic cells was assessed with an Array Scan VTI. The results are expressed as the percentage of TUNEL-positive cells. ns indicates a non-significative *p*-value, * indicates a *p*-value < 0.05, ** indicates a *p*-value < 0.01, and *** indicates a *p*-value < 0.001 by one-way ANOVA followed by Dunnett’s post hoc test for comparison of the control treatment with the other treatments.

To characterize the ER-dependent proliferative effects of these two compounds, we first analyzed their impact on MCF-7 cell number (Figure 4). Cells were treated with various doses of zearalenone (Figure 4A, solid lane) or apigenin (Figure 4B, solid lane) for 6 days. Zearalenone induced proliferation starting at 10^−10^ M, and the effect reached a plateau at approximately 80% of the effect of 10^−9^ M E2. Apigenin induced a slight increase in cell number starting at 10^−6^ M and reaching a maximum at 10^−5^ M, and the cell number represented only 50% of the cell number observed after treatment with 10^−9^ M E2. In a second experiment, cells were cotreated with a single concentration of E2 (10^−9^ M) and various doses of zearalenone (Figure 4A, dotted line) or apigenin (Figure 4B, dotted line). Under these conditions, zearalenone had no effect on the proliferation induced by 10^−9^ M E2. In contrast, apigenin showed an antagonistic effect, with a reduction in cell number to approximately 65% of that with E2 treatment only, at 10^−5^ M. Since cell number is the result of cell proliferation and cell death, we assessed the effect of treatment with 10^−8^ M zearalenone and 10^−5^ M apigenin on the cell cycle (Figure 4C) and apoptosis (Figure 4D). The cell cycle was analyzed by flow cytometry after propidium iodide staining, and the percentage of cells in each cell cycle phase was determined. As expected, E2 induced the entry of cells into the cell cycle, with a significant increase in the percentage of cells in the S and G2/M phases compared to that of control cells. Zearalenone and apigenin also promoted the entry of cells into the cell cycle by significantly increasing the percentage of cells in the S phase. However, the percentage of cells in the G2/M phase was not significantly increased compared to that of the control cells (Figure 4C). Apoptosis was determined by a TUNEL assay (Figure 4D). As described in numerous studies, E2 treatment significantly reduced the percentage of TUNEL-positive cells. Zearalenone and apigenin also reduced the percentage of TUNEL-positive cells, but this reduction was not significant compared to the percentages of TUNEL-positive control cells or E2-treated cells. In parallel, we examined the effects of zearalenone and apigenin alone and in combination with estradiol on the proliferation of T47D ER-positive breast cancer cells. Using this model, we confirmed the partial antagonistic effect of apigenin on the E2-dependent proliferation of T47D, while zearalenone alone or in combination with estradiol showed a full agonistic effect (Figure S2).

### 3.5. Genome Wide Microarray Analysis 

**Figure 5:**
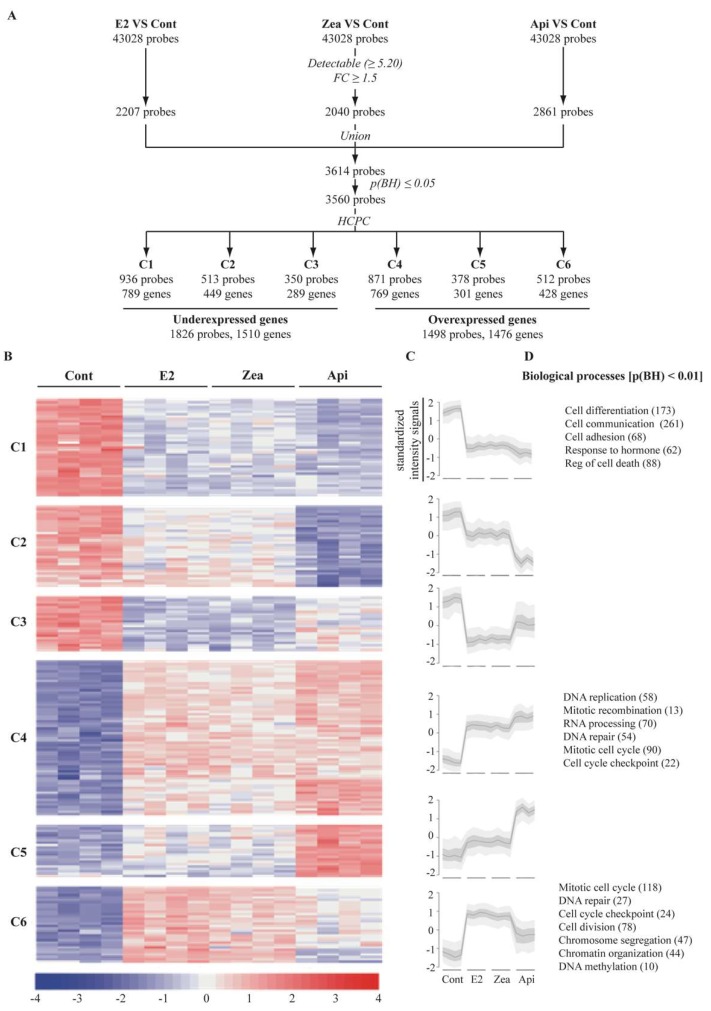
Transcriptomic analysis, selection and clustering of differentially expressed genes. MCF-7 cells were treated with solvent (Cont), 10^−9^ M E2, 10^−8^ M zearalenone (Zea) or 10^−5^ M apigenin (Api) for 24 h. Total RNA was extracted, reverse transcribed, labeled and spotted onto a DNA chip. (**A**) To select differentially expressed genes, each treatment was compared to the control, and all probes with both an intensity signal above the overall median and a fold change ≥ 1.5 were chosen. Then, the probes were combined and submitted to a LIMMA test; only probes with a *p*-value < 0.05 were selected, resulting in a total of 1510 underexpressed genes and 1498 overexpressed genes. (**B**) These genes were clustered into 6 clusters depending on their expression patterns. (**C**) Intensity signals were standardized, and the expression profile of each cluster is presented. Major biological processes significantly associated (*p*-value < 0.01) with the different clusters are noted (**D**). The number of differentially expressed genes corresponding to each gene ontology (GO) term are indicated in parentheses.

To gain more insight into the gene networks differentially regulated by zearalenone and apigenin and to determine how these compounds may influence the cellular phenotypes, a genome-wide microarray analysis was performed. Cells were treated with solvent as a control, 10^−9^ M E2, 10^−8^ M zearalenone or 10^−5^ M apigenin. The data were deposited in gene expression omnibus (GEO) (GSE1195) and in TOXsIgN (TSP763) [26]. After the statistical filtration analysis as described in the materials and methods section and in Figure 5A, 3560 probes showing a significant differential expression were selected and further classified into six expression clusters (termed C1-6). The C1-3 clusters are associated with genes (1826 probes corresponding to 1510 genes) showing a downregulated expression pattern as compared to the control samples, while C4-C6 for the three tested compounds contain genes (1498 probes, 1476 genes) significantly induced after the exposure to E2, zearalenone and apigenin (Figure 5A and Table S1). Of particular interest was that, at the dose tested, E2, zearalenone and apigenin altered the expression of the same set of genes (Figure 5B,C). Nevertheless, while E2 and zearalenone had a very similar impact on the transcriptional program of MCF-7 cells by affecting genes at similar expression level (as shown in Figure 5B,C), the level of over- or under-expression induced by apigenin is always weaker (C3 and C6) or stronger (C2, C4 or C5) than those induced by E2. To deepen the analysis, we performed a functional analysis by identifying, the major biological processes associated with those different clusters are shown in Figure 5D. Importantly, we found that underexpressed gene clusters (C1) to be significantly enriched in genes involved in cell differentiation and cell communication, while overexpressed genes clusters (C4 and C6) were mainly linked to cell cycle and cell growth. 

### 3.6. Regulatory Network Analysis Revealed

**Figure 6:**
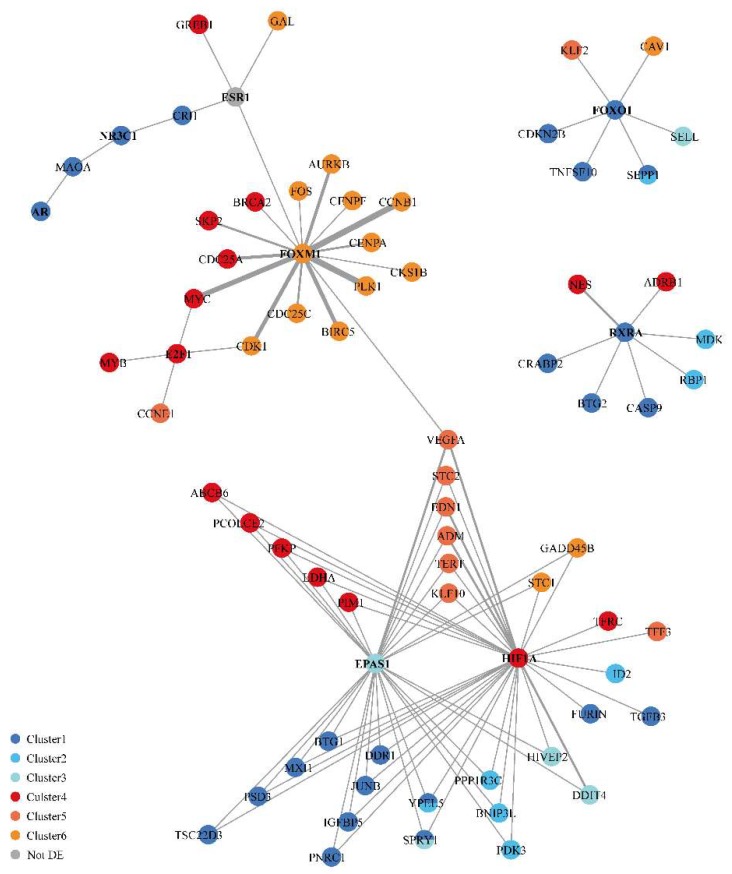
Gene regulation network built from the transcript profiling data and regulation data.

A gene-protein interaction network was built with all the differentially expressed genes (Figure 6 and Table S1), to which ERα was added. This network showed a large network composed of three communities centered on 7 transcription factors. A small community was composed of the ERα (ESR1), glucocorticoid receptor (GR, NR3C1) and androgen receptor (AR) genes, which were globally downregulated. The second community was centered on forkhead box M1 (FOXM1), which is a master regulator of several genes involved in the cell cycle, and on E2F transcription factor 1 (E2F1). These genes were upregulated. The third community was linked to hypoxia inducible factor 1 alpha (HIF1α)/ endothelial PAS domain protein 1 (EPAS1, HIF2α). These two transcription factors are well-described as key factors in controlling a responsive gene network during hypoxia or as being involved in cell growth arrest or metabolic reprogramming. In this community, half of the genes were upregulated, and half were downregulated. Two other small networks were also defined. One was centered on the transcription factor forkhead box O1 (FOXO1), which was downregulated. The other was centered on retinoid X receptor alpha (RXRA), which was also downregulated. 

Each gene/protein is represented by a node color-coded according to the related expression clusters. The edges between the nodes correspond to the protein/DNA interactions, and the thickness of each edge is linked to the number of publications describing this interaction. Transcription factors are indicated in bold. 

### 3.7. Zearalenone and Apigenin alter the Expression of Genes Involved in Cell Cycle and Growth Arrest

**Figure 7:**
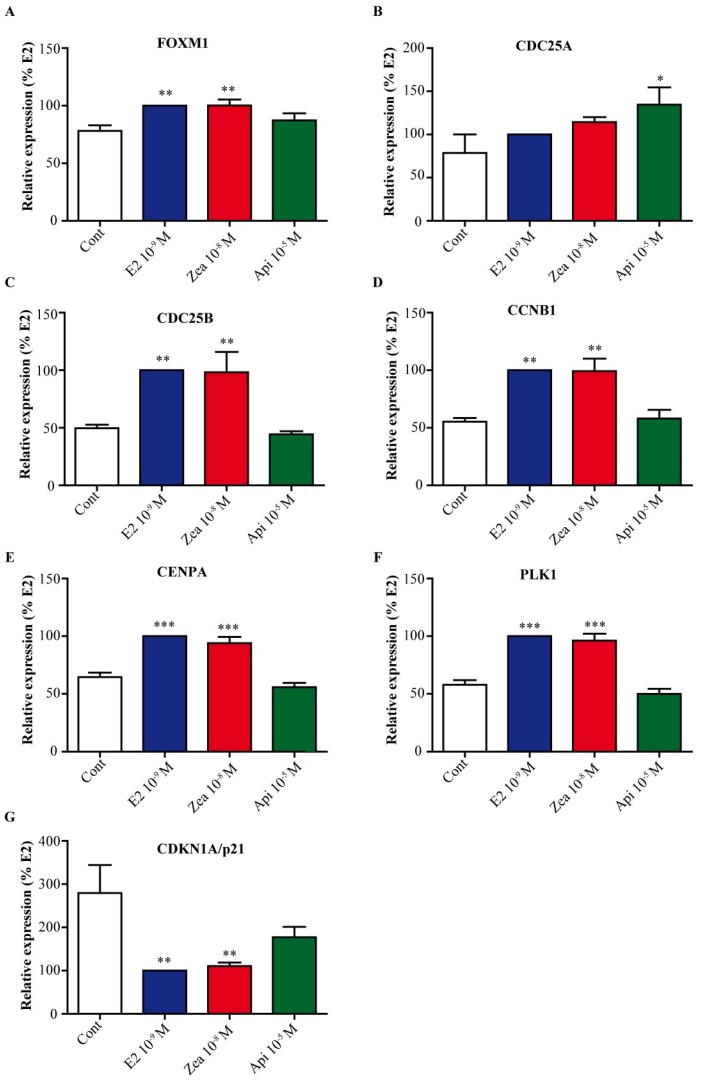
Validation of cell cycle-associated genes linked to forkhead box M1 (FOXM1). MCF-7 cells were treated with solvent (white) as a negative control, 10^−9^ M E2 (blue) as a positive control, 10^−8^ M zearalenone (red) or 10^−5^ M apigenin (green) for 24 h. The expression of FOXM1 (**A**), cell division cycle 25A (CDC25A) (**B**), cell division cycle 25B (CDC25B) (**C**), cyclin B1 (CCNB1) (**D**), centromere protein A (CENPA) (**E**), polo like kinase 1 (PLK1) (**F**) and cyclin dependent kinase inhibitor 1A (CDKN1A/p21) (**G**) was assessed by real-time PCR. The expression level of each gene was normalized to the expression levels of the housekeeping genes glyceraldehyde-3-phosphate dehydrogenase (GAPDH) and TATA box binding protein (TBP). The results are expressed as the percentage of relative expression of transcripts obtained in E2-treated cells and are the means ± SEM of six independent experiments. * indicates a *p*-value < 0.05, ** indicates a *p*-value < 0.01 and *** indicates a *p*-value < 0.001 by one-way ANOVA followed by Dunnett’s post hoc test for comparison of the control treatment with the other treatments.

In cluster 6 and for the most part in cluster 4, gene ontology (GO) terms associated with the cell cycle were significantly (*p* < 0.01) enriched. Notably, the transcription factor FOXM1, which was differentially expressed, controls the expression of numerous genes involved in cell cycle progression. Thus, we first validated our transcriptomic data for several genes involved in cell cycle progression, such as FOXM1 (Figure 7A), cell division cycle 25A (CDC25A) (Figure 7B), cell division cycle 25B (CDC25B) (Figure 7C), cyclin B1 (CCNB1) (Figure 7D), centromere protein A (CENPA) (Figure 7E), polo like kinase 1 (PLK1) (Figure 7F) and cyclin dependent kinase inhibitor 1A CDKN1A (p21^cip1^) (Figure 7G). Compared to their levels in control cells, genes belonging to cluster 4 (FOXM1, CDC25B, CCNB1, CENPA and PLK1) were significantly upregulated (*p* < 0.01) by 10^−9^ M E2 and 10^−8^ M zearalenone, while apigenin 10^−5^ M did not affect the expression of these genes. CDC25A belonged to cluster 6 and was slightly but not significantly upregulated by E2 and zearalenone, while apigenin upregulated CDC25A expression significantly (*p* < 0.01). Finally, CDKNA1, which is involved in cell cycle arrest, was significantly downregulated (*p* < 0.01) by E2 and zearalenone and was slightly but not significantly downregulated by apigenin.

**Figure 8:**
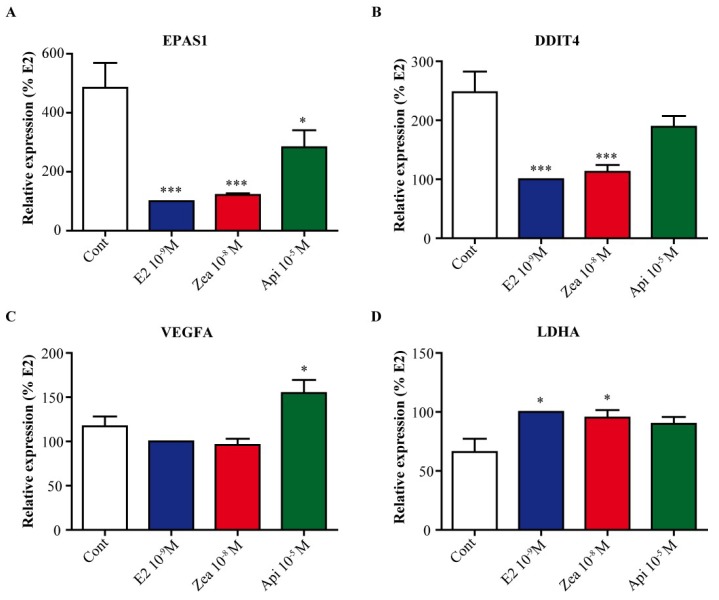
Validation of metabolism and growth arrest genes linked to HIF1α and EPAS1/HIF2α.MCF-7 cells were treated with solvent (white) as a negative control, 10^−9^ M E2 (blue) as a positive control, 10^−8^ M zearalenone (red) or 10^−5^ M apigenin (green) for 24 h. The expression of EPAS1/HIF2α (**A**), DNA damage inducible transcript 4 (DDIT4) (**B**), vascular endothelial growth factor A (VEGFA) (**C**) and lactate dehydrogenase A (LDHA) (**D**) was assessed by real-time PCR. The expression level of each gene was normalized to the expression levels of the housekeeping genes GAPDH and TBP. The results are expressed as the percentage of the relative expression of transcripts obtained in E2-treated cells and are the means ± SEM of six independent experiments. * indicates a *p*-value < 0.05 and *** indicates a *p*-value < 0.001 by one-way ANOVA followed by Dunnett’s post hoc test for comparison of the control treatment with the other treatments.

Another community in the network was centered on the transcription factors HIF1α and EPAS1/HIF2α. These transcription factors are involved in the response to hypoxia not only by inducing genes such as vascular endothelial growth factor A (VEGFA) and lactate dehydrogenase A (LDHA) but also by regulating growth arrest genes such as DNA damage inducible transcript 4 (DDIT4). As shown in Figure 8A, E2 and zearalenone strongly downregulated the expression of the transcription factor EPAS1/HIF2α (by 4 times; *p* < 0.001) while apigenin resulted in a reduction of only 2 times (*p* < 0.05). Of particular interest was that DDIT4, which is an inhibitor of mammalian target of rapamycin (mTOR) and is involved in cell growth, was significantly downregulated by E2 and zearalenone (*p* < 0.001) but not by apigenin (Figure 8B). On the other hand, the expression of VEGFA, which was significantly induced by apigenin (*p* < 0.05), was not modified by E2 or zearalenone (Figure 8C), while the expression of LDHA was significantly induced by E2 and zearalenone (*p* < 0.05) but not by apigenin (Figure 8D). Differential regulation of DDIT4 gene expression was also confirmed in T47D cells (Figure S1C).

### 3.8. Zearalenone and Apigenin Alter the Expression of Genes Linked to Cancer, Nucleoli and Apoptosis

**Figure 9:**
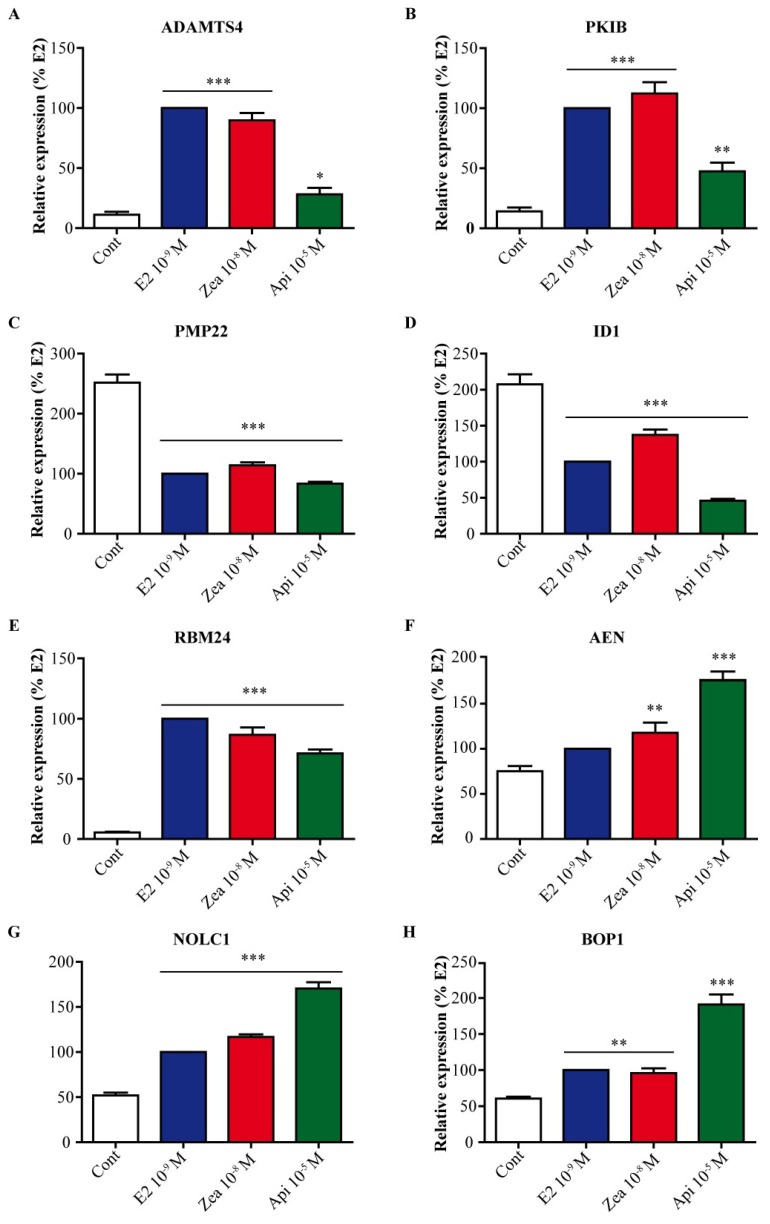
Validation of genes linked to cancer, nucleoli and apoptosis. MCF-7 cells were treated with solvent (white) as a negative control, 10^−9^ M E2 (blue) as a positive control, 10^−8^ M zearalenone (red) or 10^−5^ M apigenin (green) for 24 h. The expression of ADAM metallopeptidase with thrombospondin type 1 motif 4 (ADAMTS4) (**A**), CAMP-dependent protein kinase inhibitor beta (PKIB) (**B**), peripheral myelin protein 22 (PMP22) (**C**), inhibitor of differentiation 1 (ID1) (**D**), RNA binding motif 24 (RBM24) (**E**), apoptosis-enhancing nuclease (AEN) (**F**), nucleolar and coiled-body phosphoprotein 1 (NOLC1) (**G**) and block of proliferation 1 (BOP1) (**H**) was assessed by real-time PCR. The expression level of each gene was normalized to the expression levels of the housekeeping genes GAPDH and TBP. The results are expressed as the percentage of the relative expression of transcripts obtained in E2-treated cells and are the means ± SEM of six independent experiments. * indicates a *p*-value < 0.05, ** indicates a *p*-value < 0.01 and *** indicates a *p*-value < 0.001 by one-way ANOVA followed by Dunnett’s post hoc test for comparison of the control treatment with the other treatments.

Furthermore, the expression of various genes in the different clusters described above was validated by real-time PCR (Figure 9). Thus, in connection with breast cancer, we examined the expression of genes encoding oncogenes that promote tumorigenesis or those associated with a poor prognosis, such as ADAM metallopeptidase with thrombospondin type 1 motif 4 (ADAMTS4), CAMP-dependent protein kinase inhibitor beta (PKIB), peripheral myelin protein 22 (PMP22), and inhibitor of differentiation 1 (ID1) [27,28,29,30]. Interestingly, ADAMTS4 and PKIB were significantly upregulated by E2 and zearalenone (*p* < 0.001) but were much less upregulated by apigenin (*p* < 0.05 and *p* < 0.01, respectively) (Figure 9A,B). We confirmed this result in T47D cells, where the expression of ADAMTS4 gene was markedly induced by E2 and zeralenone but not by apigenin (Figure S1B). On the other hand, the PMP22 and ID1 genes were significantly downregulated by E2 and zearalenone, but ID1 gene expression was more strongly downregulated by apigenin (*p* < 0.001) (Figure 9C,D). RBM24 (RNA binding motif 24) (Figure 9E) has not been described in breast cancer, but this gene was characterized to suppress nasopharyngeal carcinoma progression [31]. In addition, RBM24 has an essential role in mediating the proper expression of p53 [32]. Our work shows for the first time that the RBM24 gene is significantly upregulated by E2, zearalenone and apigenin (*p* < 0.001), suggesting that RBM24 may be a new ER target gene in breast cancer. In cluster 5, AEN (apoptosis-enhancing nuclease) (Figure 9F), which is associated with p53-mediated apoptosis, was significantly upregulated by zearalenone and apigenin (*p* < 0.01 and *p* < 0.001, respectively) suggesting a potential stress induced by these two compounds. Moreover, NOLC1 (nucleolar and coiled-body phosphoprotein 1) (Figure 9G) as well as BOP1 (block of proliferation 1) (Figure 9H) are involved in RNA processing in the nucleoli. Thus, it could be interesting to consider that apigenin upregulated these two genes more strongly than E2 and zearalenone did. 

## 4. Discussion

Breast cancer is a major public health concern with an estimated economic cost of €15 billion in the European Union [33]. The etiology of breast cancer is complex, with multiple causes (genetic, environmental aggression, etc.) and large heterogeneity. Dietary is an important part of our environment. It is now well established that diet acts on our health. For instance, a recent study showed that organic food consumption reduced cancer risk [34], even if this study needs to be confirmed. Zearalenone and apigenin are found in the diet and, consequently, may impact the etiology of breast cancer; zearalenone is an exogenous toxin, and apigenin is an endogenous compound in numerous plant foods. Using different approaches, we and others showed that these two compounds are able to bind and activate ERs [5,35,36,37,38]. Recently, we showed that zearalenone and apigenin have differential effects on ERα-mediated proliferation and differentiation [5]. In this work, we studied the molecular mechanisms sustaining the activity of apigenin and zearalenone on ER-positive MCF-7 breast cancer cells proliferation. 

Our results confirmed that zearalenone and apigenin activate ERα on the ERE-controlled luciferase reporter gene, although a difference in the kinetics of luciferase activity was observed. ChIP experiments showed no major differences in ERα recruitment to various ER binding sites when ERα was activated by zearalenone or apigenin. However, zearalenone elicited a full and powerful proliferative effect on breast cancer cells in both the absence and presence of E2, while apigenin showed a partial and weak proliferative effect in the absence of E2 and an antagonistic effect in the presence of E2. We showed these effects in both ER-positive breast cancer cell lines, MCF-7 and T47D. Taken together, these results suggest that apigenin is a partial agonist, while zearalenone is a full ERα agonist. 

ERα has two transactivation domains; AF-1 is located in the N-terminus and is ligand-independent, whereas AF-2 is in the C-terminus in the ligand-binding domain (LBD). AF-2 contains a flexible region corresponding to the helix 12 which is important for the recruitment of co-factor. The binding of E2 to ERα gives rise to a conformational change in ERα which allows the active positioning of the helix 12 in AF2 transactivation and a synergistic effect between AF-1 and AF-2 for the recruitment of coactivators such as SRC-1 [39]. ER structure-function studies have reported that certain estrogen-like compounds allow the establishment of a conformation distinct from that induced by E2. This remarkable property for these molecules would offer promising therapeutic prospects in E2-associated diseases. In a recent work using different AF-2 mutants of ERα, Arao et al. showed that zearalenone and E2 induced a conformation of ERα that fully activated ERα even with the mutation of the static region of AF-2. Conversely, apigenin was not able to induce ERα bearing a mutation in AF-2, suggesting that the conformation of the LBD was not optimal [36,40]. Moreover, docking experiments of zearalenone and apigenin to the LBD of ERα showed a real difference in docking between these compounds. Indeed, it was demonstrated that the interaction of ligands with His-524 was necessary for full agonist activity [41]. Zearalenone interacts with His-524, which permits the proper folding of helix 12 and the recruitment of coactivators [38,42]. In contrast, apigenin interacts very weakly or not at all with His-524 [37]. To obtain a global insight into the effect of these compounds on ER-mediated transcription that could also explain their differential effects on cellular fate, a genome-wide microarray analysis of MCF-7 breast cancer cells was performed. Interestingly, this analysis demonstrated that both compounds regulate the expression of ER-target genes in the same direction but at different intensities, which could be explained by the differential interaction of zearalenone and apigenin with the LBD of ERα. As suggested previously, depending on the chromatin context, including the presence of epigenetic marks, the mechanism of binding to an ERE directly or by tethering (via AP-1 and SP-1) and the concentration of cofactors, the intensity of ER target gene regulation could be different [43]. Our transcriptomic analysis revealed two regulatory networks, namely, a hub centered on FOXM1 and ERα and a hub centered on HIF1α/EPAS1, as also found in a previous work on the glyceollin phytoalexins [20]. This result indicates the existence of common links between these molecules and molecular pathways for the growth and apoptosis of breast cancer cells. Indeed, FOXM1 is a key regulator of several genes involved in cell cycle progression, especially in the G1/S and G2/M transitions [44]. In contrast to zearalenone and E2, apigenin did not enhance the expression of FOXM1, and all tested genes except CDC25A followed the expression pattern of this transcription factor. For instance, the CDC25 family (CDC25A-C) is a new target for the treatment of triple-negative breast cancer [45]. In our study, apigenin did not induce the expression of two members of this family (CDC25B and CDC25C). A cell cycle analysis showed that all treatments induced cell entry into the G1/S phase. In accordance with this observation, CDC25A expression was induced, although only apigenin treatment resulted in significant upregulation. However, treatment with zearalenone or apigenin did not result in a significant increase in the percentage of cells in the G2/M phase compared to that of the control cells. The expression of CDC25B or CCNB1, which are involved in G2/M progression, was in accordance with this observation for apigenin but not for zearalenone, suggesting a possible induction of checkpoint control by zearalenone treatment. This hypothesis is reinforced by the slight increase in the percentage of TUNEL-positive zearalenone-treated cells compared to the percentage of TUNEL-positive E2-treated cells. 

The second hub in the regulatory network was centered on the HIF1α and EPAS1 transcription factors. This family of transcription factors is well-described for their role in adaptation to low-O_2_ conditions [46]. HIF1α was slightly increased by treatment with all compounds tested (Table S1), while EPAS1/HIF2α were clearly downregulated by E2 and zearalenone treatment and less by apigenin treatment. This result is consistent with those in our previous work and in other studies showing that estrogens upregulate the expression of HIF1α and downregulate the expression of the EPAS1/HIF2α genes [20,47,48]. Consequently, the expression of direct HIF target genes such as lactate dehydrogenase A (LDHA) or vascular endothelial growth factor A (VEGFA) was upregulated by E2 and zearalenone or by apigenin, respectively. These genes are involved in the adaptation of cells to hypoxia and to neovascularization, respectively. More importantly, the expression of DDIT4 (also known as REDD1), an inhibitor of mTOR [49], was significantly downregulated by E2 and zearalenone but not by apigenin. mTOR is a downstream effector of the PI3K/AKT pathway, which regulates protein synthesis by promoting the phosphorylation of p70S6K. 

In parallel with the direct effect of treatment on gene expression, the impact of apigenin and zearalenone on intracellular pathways might explain their proliferative or antiproliferative activity. For example, the inhibition of the PI3K/AKT/mTOR pathway by apigenin was described in several cancers, including prostate cancer [50], lung cancer [51] and hepatocellular carcinoma [52]. Another major pathway involved in the proliferation of breast cancer cells is the MAPK pathway, which is involved particularly via a rapid and transient interaction between ERα and the intracellular SRC proto-oncogene, non-receptor tyrosine kinase (c-Src/Src) [16]. Using a proximity ligation assay, we observed a slight increase in ERα/Src interaction in response to E2 and zearalenone treatment, while apigenin treatment weakly but not significantly repressed this interaction (Figure S3). The decrease in this interaction was accompanied by a weak decrease in the phosphorylation of Src after 30 min (Figure S4A) and an increase in p53 phosphorylation after 24 hours of apigenin treatment compared to E2 (Figure S4B) observed with the proteome profiler kit. These results on the regulation of gene expression and the modulation of intracellular pathways suggest that apigenin induces a senescence phenotype characterized by the sustained expression of growth arrest genes and by the induction of the p53 pathway, as confirmed by the induction of AEN or RBM24.

## 5. Conclusions

In this study, we sought to decipher molecular mechanisms sustaining the estrogenic activity of zearalenone and apigenin in an ER-positive breast cancer cell model, and to better understand how these compounds could differentially affect cell fate. This study, like others before, confirmed the selective estrogen receptor modulator (SERM) potential of apigenin in ER-positive breast cancer and did not contest its potential health benefit effect. However, a drawback concerning the use of apigenin is its low bioavailability. It will be interesting to increase this bioavailability by chemical functionalization or galenical approaches. For instance, in one study, the use of a carbon nanopowder succeeded in increasing the bioavailability of apigenin [53]. In contrast, zearalenone appears to be a true endocrine-disrupting compound that may contribute to tumorigenesis even at very low doses, as was suggested in a recent review [54]. 

## Figures and Tables

**Table 1:** Gene names and primer sequences used in the real-time polymerase chain reaction (PCR) experiments.

Gene Name and Symbol	Forward Primer	Reverse Primer
Chemokine (C-X-C motif) ligand 12 (CXCL12)	CACCATTGAGAGGTCGGAAG	AATGAGACCCGTCTTTGCAG
Progesterone receptor (PgR)	CCCGCCGTCGTAACTTTGG	GTGCCTATCCTGCCTCTCAATC
Amphiregulin (AREG)	GTATTTTCACTTTCCGTCTTGTTTTG	CCTGGCTATATTGTCGATTCA
Growth regulation in breast cancer 1 (GREB1)	GAGGATGTGGAGTGGAGACC	CAGTACCTCAAAGACCTCGGC
Forkhead box M1 (FOXM1)	AGCGAGACCCATCAAAGTGG	GGTCTTGGGGTGGGAGATTG
Cell division cycle 25A (CDC25A)	CAAGGGTGCAGTGAACTTGC	ACAACAATGACACGCTTGCC
Cell division cycle 25B (CDC25B)	CTACTGCTGTGAACCCTGGG	CAACAAAACGCTCCCACCTG
Cyclin B1 (CCNB1)	TCTGGATAATGGTGAATGGACA	CGATGTGGCATACTTGTTCTTG
Centromere protein A (CENPA)	ACATGCAGGCCGAGTTACTC	AGAGTCCCCGGTATCATCCC
Polo-like kinase 1 (PLK1)	CTCAACACGCCTCATCCTC	GTGCTCGCTCATGTAATTGC
Cyclin-dependent kinase inhibitor 1A (CDKN1A/p21)	CTGTCTTGTACCCTTGTGCC	GGTAGAAATCTGTCATGCTGG
Endothelial PAS domain protein 1 (EPAS1/HIF2α)	GCGCTAGACTCCGAGAACAT	TGGCCACTTACTACCTGACCCTT
Vascular endothelial growth factor A (VEGFA)	AGGAGGAGGGCAGAATCATCA	CTCGATTGGATGGCAGTAGCT
Lactate dehydrogenase A (LDHA)	GGCCTGTGCCATCAGTATCT	GCCGTGATAATGACCAGCTT
DNA damage-inducible transcript 4 (DDIT4/REDD1)	AGGAAGCTCATTGAGTTGTG	GGTACATGCTACACACACAT
Glyceraldehyde 3-phosphate dehydrogenase (GAPDH)	GGGCATCCTGGGCTACACTG	GGGCATCCTGGGCTACACTG
TATA box binding protein (TBP)	TGCACAGGAGCCAAGAGTGAA	CACATCACAGCTCCCCACCA

## Supplementary Materials

The following are available online at http://www.mdpi.com/2072-6643/11/2/237/s1, Table S1: All the genes differentially expressed found in the transcriptomic analysis were presented with their names, their gene ontologies, and their regulations, Figure S1: T47D cells were treated with solvent (white) as a negative control, 10^−9^ M E2 (blue) as a positive control or with 10^−8^ M zearalenone (Zea, red) or with 10^−5^ M apigenin (Api, green) for 24 h. The expression of CXCL12 (A), ADAMTS4 (B), DDIT4 (C), CDC25B (D) and BOP1 (E) was assessed by real-time PCR. The expression level of each gene was normalized to the expression levels of the housekeeping genes GAPDH and TBP. The results are expressed as the percentage of the relative expression of transcripts obtained in E2-treated cells and are the means ± SEM of at least three independent experiments, Figure S2: T47D cells were grown for 6 days in serum-free medium in the presence of solvent (white), 10^−9^ M E2 (blue), 10^−8^ M Zea (red) and 10^−5^ M Api (green) alone or in combination with 10-9 M E2 (hatched). Cell number was determined by counting. The results are expressed as the percentage of the cell number counted after E2 treatment and are the means ± SEM of three experiments, Figure S3: Number of ERα/c-SRC interaction /cell after a treatment of 5 minutes with solvent as negative control (white), 10^−9^ M E2 (blue), 10^−8^ M zearalenone (Zea, red) or 10^−5^ M apigenin (Api, green). Interactions were monitored by Proximity Ligation Assay according to manufacturer instruction using anti-ERα (HC-20) (Sc-543) and anti-cSRC (B-12) (Sc-8056) antibodies (Santa Cruz Biotechnology). Results are expressed percentage of controls and are the mean +/- SEM of 3 independent experiments, Figure S4: Analysis of Src (A) and p53 (B) phosphorylation using the Proteome Profiler Kit according to the manufacturer instruction. For Src, cells were treated with solvent as negative control, 10^−9^ M E2, 10^−8^ M Zea or 10^−5^ M Api for 30 min. For the different p53 phosphorylation, cells were treated with 10^−9^ M E2 or 10^−5^ M Api for 24 h. Results are expressed as the relative intensity of specific phosphorylation forms compared to reference spot in arbitrary unit.
