# Supplementary figures and images for: Deciphering the Molecular Mechanisms Sustaining the Estrogenic Activity of the Two Major Dietary Compounds Zearalenone and Apigenin in ER-Positive Breast Cancer Cell Lines
Source: Nutrients. 2019 Jan 22;11(2):237. doi: 10.3390/nu11020237 (PMC6412274; doi:10.3390/nu11020237)

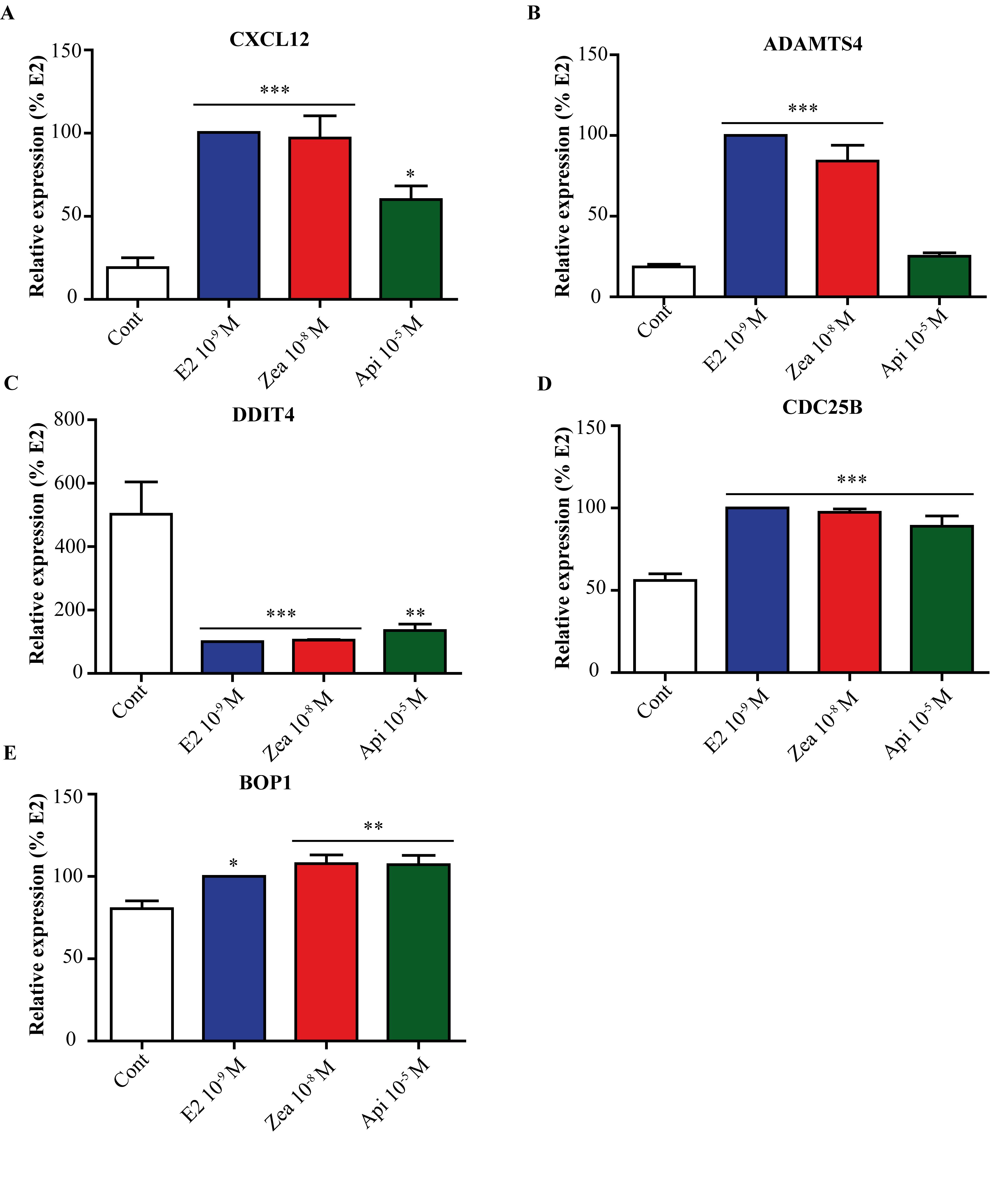

Supplement: Supplementary file 1 [file nutrients-11-00237-s001.zip › Supl-fig-397052/Figure S 1 .tif]

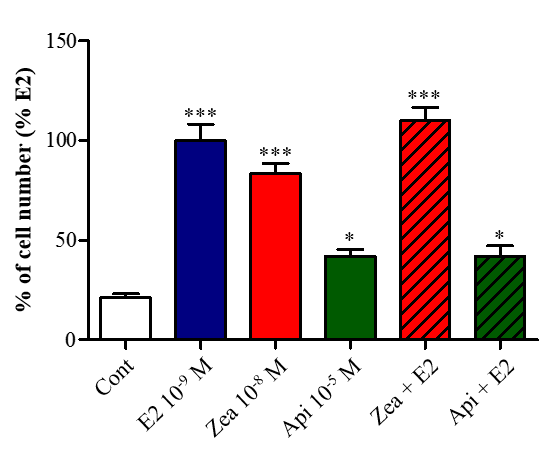

Supplement: Supplementary file 1 [file nutrients-11-00237-s001.zip › Supl-fig-397052/Figure S2.tif]

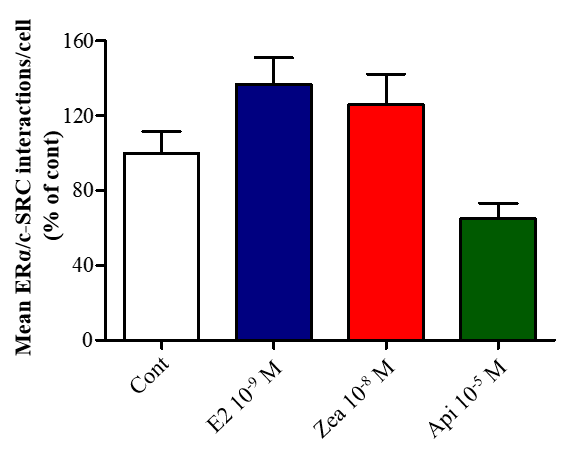

Supplement: Supplementary file 1 [file nutrients-11-00237-s001.zip › Supl-fig-397052/Figure S3.tif]

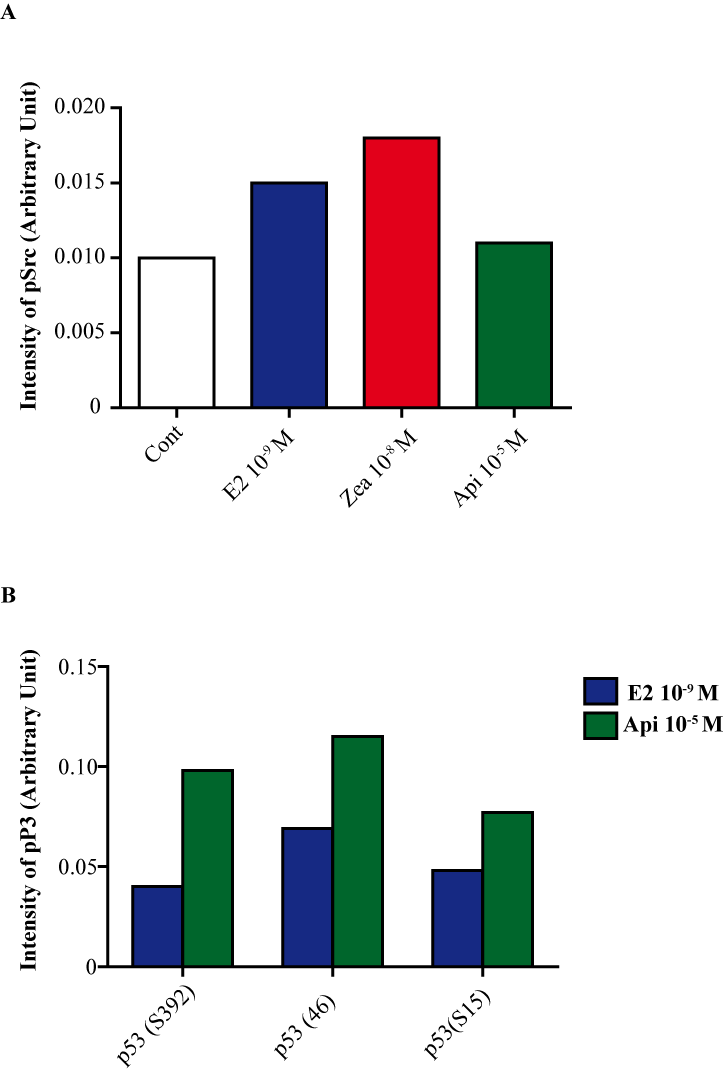

Supplement: Supplementary file 1 [file nutrients-11-00237-s001.zip › Supl-fig-397052/Figure S4.png]
